# Association of PD-1/PD-L1 expression and Epstein-–Barr virus infection in patients with invasive breast cancer

**DOI:** 10.1186/s13000-022-01234-3

**Published:** 2022-07-16

**Authors:** Wei-tong Zhang, Gui-lu Zhu, Wu-qin Xu, Wei Zhang, Hui-zhen Wang, Ya-bing Wang, Yong-xiang Li

**Affiliations:** 1 Department of General Surgery, The First Affiliated Hospital of Anhui Medical University, Hefei, 230022 China; 2 Department of Clinical Pathology, Yijishan Hospital, Wannan Medical College, Wuhu, 241001 China; 3 Breast Surgery, Yijishan Hospital, Wannan Medical College, Wuhu, 241001 China

**Keywords:** Breast cancer, Epstein-Barr virus, Immunotherapy, PD-1, PD-L1

## Abstract

**Purpose:**

Causative factors of breast cancer include infections, such as Epstein–Barr virus (EBV) infection. The aim of this study was to analyze the clinicopathological features of EBV-positive (IBC) and determine if EBV affects programmed cell death receptor 1 (PD-1)/PD ligand 1 (PD-L1) expression in IBC, similar to other EBV-infected tumors with PD-L1/PD-1 expression.

**Methods:**

We collected 140 samples of IBC tissues and 25 samples of adjacent tissues. All patients were followed-up by telephone from the day of surgery to December 2020. Chromogenic in-situ hybridization was performed to evaluate EBV-encoded RNA (EBER). Immunohistochemistry was performed to evaluate PD-L1 and PD-1 expressions. The correlation between PD1/PDL1 expression and clinicopathological features was also analyzed.

**Results:**

EBER was detected in 57 of 140 (40.7%) IBC tissues and not detected in any adjacent tissue (*P* < 0.05). Clinicopathologic features of patients were consistent with EBV-associated IBC. EBV infection was correlated with the mass size, menopausal status, axillary lymph node metastasis, vascular invasion, Ki-67 index, clinical stage, and estrogen receptor and progesterone receptor expressions (all *P* < 0.05), but not with the histological type, invasive ductal carcinoma histological grade, or human epidermal growth factor receptor 2 (HER2) expression (all *P* > 0.05). The positive rate of PD-1/PD-L1 expression was higher in the EBV-positive group than in the EBV-negative group (*P* < 0.05). The Kaplan–Meier univariate survival analysis showed that EBV was associated with poor disease-free survival and overall survival in patients with IBC. PD-L1/PD-1 expression could predict a poor prognosis.

**Conclusions:**

In this study, clinicopathologic characteristics of patients were consistent with EBV-infected IBC. Patients with EBV-positive breast cancer were more likely to have elevated PD-1/PDL-1 expression compared to those with EBV-negative breast cancer. This finding could serve as a basis to explore therapeutic targets, particularly immunotherapy, for patients with IBC.

## Summary

We collected 140 samples of invasive breast cancer (IBC) tissues and 25 samples of adjacent tissues. Chromogenic in-situ hybridization showed that some patients with IBC had Epstein–Barr virus (EBV) infection based on EBV-encoded RNA. Patients in the study had a large tumor mass, postmenopausal status, frequent axillary lymph node metastasis and lymphovascular invasion, high clinical stage, and generally negative estrogen and progesterone expressions, all of which are consistent with EBV-infected IBC. Patients with a positive rate of PD-1/PD-L1 expression were more likely to have EBV-infected breast cancer compared to those without. Patients with EBV infection also had a poor prognosis. PD-L1/PD-1 expression could also predict a poor prognosis. These findings could serve as a basis to explore therapeutic targets in these patients.

## Introduction

The cause of breast cancer has not been established. The known risk factors include reproductive factors, affecting the balance of sex hormones in the body, and genetic susceptibility, but do not account for all breast cancer cases. In recent years, viral infections have been investigated as a cause of breast cancer.

The Epstein–Barr virus (EBV) is a human herpes virus that causes lymphocytic infection. It belongs to the γ subfamily of the herpesvirus family, and its genetic material is DNA. As latent herpesvirus infection, EBV exists in the human body and can promote the transformation of human cells leading to unlimited cell proliferation. EBV infection is associated with various tumors in humans, including Burkitt’s lymphoma, nasopharyngeal carcinoma, and gastric carcinoma [1–5]. Labrecque et al. in 1995 reported that EBV was associated with breast cancer, and the correlation between EBV and breast cancer has since been studied [6]. However, the results have been controversial.

Owing to the progress in research of the immunomodulatory signaling pathway in immune cells and tumor microenvironment, targeted drugs, particularly immune checkpoint inhibitors, have been introduced in clinical practice with good results. Currently, major immune checkpoints in breast cancer include programmed cell death receptor 1 (PD-1)/PD ligand 1 (PD-L1) and cytotoxic T lymphocyte antigen-4. Dong et al. in 1999 searched the human gene pool for B7–1 and B7–2 immunoglobulin variable and constant region homologues and found PD-L1 in the placental cDNA library [7]. PD-L1 is also called B7-H1 (or “B7-homolog 1,” “B7 homolog1,” and “B7 molecular homolog1 protein”) and CD274. Its gene is located on chromosome 9p24. PD-1 is a receptor of PD-L1, and its gene is located on chromosome 2q37. PD1 combined with PD-L1 can inhibit the proliferation and activation of CD4- and CD8-positive T cells, downregulate the secretions of interleukin-2 and interferon-γ, and finally inhibit the immune function of T cells, thereby forming an immunosuppressive tumor microenvironment in which tumor cells escape the host’s immune system. Blocking the PD1/PD-L1 signal transduction pathway can reverse the immunosuppressive tumor microenvironment, restore the antitumor activity of T cells, and enhance the antitumor effect of the host’s immune system [8].

In various cancer caused by EBV infection, including EBV-infected nasopharyngeal cancer, gastric cancer, and classic Hodgkin lymphoma, the PD-L1/PD-1 expression is further increased [9–12]. Whether this phenomenon exists in breast cancer caused by EBV infection or not is unknown.

The aim of the present study was to analyze the clinicopathological features of EBV-positive (IBC) and the relationship between PD-L1/PD-1 expression and EBV infection in IBC. The correlation between PD1/PDL1 expression and clinicopathological parameters was also analyzed. Finally, we discuss the relevance of EBV infection in the occurrence and development of breast cancer to further improve the clinical understanding of breast cancer prevention, diagnosis, prognosis evaluation, and treatment.

## Materials and methods

### Samples

This retrospective study involved a total of 140 specimens of IBC obtained from patients without neoadjuvant chemotherapy from the Department of Breast Surgery, the First Affiliated Hospital of Wannan Medical College, from June 2006 to January 2020, and 25 specimens of the adjacent healthy tissues. Clinical details of the patients were obtained from electronic medical records. All patients were followed-up by telephone from the day of surgery to December 2020, with distant metastasis or death as the endpoints. Twenty-six patients were lost to follow-up.

### Histopathological evaluation

All tissues were fixed with 10% neutral buffered formalin, embedded in paraffin, and observed after hematoxylin & eosin staining, immunohistochemical staining, and in situ molecular hybridization in 4-μm serial sections. Two breast pathologists determined the histological types in accordance with the WHO Classification of Breast Tumors, 5th edition [13], and histological grade on the Scarff–Bloom–Richardson grading system [13]. The molecular classification of IBC was performed as described by Sorlic et al [14]

### IBC tissue EBER-CISH

The CISH apparatus was ThermoBrite StatSpin (IRIS International Int., USA). The EBER in situ hybridization kit was purchased from Leica Biosystems Newcastle Ltd., Germany. The paraffin sections were dewaxed with xylene, hydrated with gradient ethanol, digested with gastric enzymes, and hybridized with digoxin-labeled EBER oligonucleotide probes, including the antisense probe 5′-AGACACCGTCCTACCACCCGGGACTTGTA-3′ and sense probe 5′-TCTGTGGCAGGAGTGGTGGGCCCTGAACAT-3′. The remaining probes were washed off from the paraffin sections and incubated with alkaline phosphatase-labeled digoxin antibody. The sections were counterstained with hematoxylin and sealed with neutral gum. Microscopic observation was performed for the EBER expression in the nucleus. EBER-positive nuclei were stained brownish yellow, while EBER-negative nuclei were unstained.

### Immunohistochemistry

Immunohistochemical staining was performed on the automated Leica BOND-MAX immunohistochemical apparatus. PD-L1 (clone SP142) and PD-1 (clone NRQ-22) were purchased from Beijing Zhongshan Jinqiao Biotechnology Co., Ltd. The experiment was carried out in strict accordance with the manufacturer’s instructions. Phosphate-buffered saline was used instead of the primary antibody as the negative control, and tonsil tissue was used as the positive control. Two blinded breast pathologists independently reviewed immunohistochemical results. Scoring was based on PD-L1-expressing tumor-infiltrating immune cells (IC) as a percentage of tumor area: IC negative (< 1%) or positive (≥1%). PD-L1 scoring on tumor cells (TC) was based on the percentage of PD-L1–expressing TC: TC negative (< 1%) or TC positive (≥1%) [15] . PD-1 was expressed in tumor-infiltrating lymphocytes (TILs). Specimens with ≥5% membranous expression were considered to be “positive” [16].

Immunohistochemistry was used to detect hormone receptors (estrogen and progesterone receptors), human epidermal growth factor receptor 2 (HER2), and Ki-67. For equivocal immunohistochemistry results, in-situ hybridization was used to confirm the HER2 status [17]. For Ki-67, the percentage score was determined using immunohistochemistry with a 14% tangent [18].

### Statistical analysis

Statistical analyses were performed using SPSS 22.0 for Windows. The χ2 test to analyze the association between clinicopathological data and EBV infection. The survival analysis was performed with the Kaplan–Meier method. Statistical significance was set at a *P*-value < 0.05.

## Results

### Clinicopathological characteristics of the cohort

Table 1 summarizes the clinical and pathologic characteristics of all patients. We enrolled 140 patients with IBC with a median age of 54 (28–82) years. The median tumor diameter was 3.5 (0.8–8.6) cm. The histological subtype was invasive ductal carcinoma in 112 (80%) cases and invasive lobular carcinoma in 28 (20%) cases, including 12 (42.9%) cases of classic lobular carcinoma and 16 (57.1%) cases of pleomorphic lobular carcinoma.

**Table 1:** Clinicopathologic characteristics of patients by EBV status

Clinicopathologic parameters	IBC tissue EBER-ISH		
	Positive	Negative	*P-value*
age			0.705
≤ 50	15 (37.5%)	25(62.5%)	
>50	42(42.0%)	58(52.0%)	
tumor size			0.043
≤ 2.0 cm	28 (33.7%)	55 (66.3%)	
> 2.0 cm	29 (50.9%)	28 (49.1%)	
Menopausal status			< 0.01
postmenopausal	22 (23.7%)	71 (76.3%)	
menstruating	35 (74.5%)	12 (25.5%)	
Histological subtype			0.302
IDC	48 (42.9%)	64 (57.1%)	
ILC	9 (32.1%)	19 (67.9%)	
Histological classification of IDC			0.22
I	7 (24.1%)	22 (75.9%)	
II	15 (42.9%)	20 (57.1%)	
III	26 (54.2%)	22 (45.8%)	
Nodal metastasis			< 0.01
Present	37 (59.7%)	25 (40.3%)	
Absent	20 (25.6%)	58 (74.4%)	
Lymphovascular Invasion			< 0.01
Present	31 (72.1%)	12 (27.9%)	
Absent	26 (26.8%)	71 (73.2%)	
Ki-67 Index			0.006
<14%	7 (21.2%)	26(78.8%)	
>14%	50(57.1%)	57(53.3%)	
TNM Clinical Stage			< 0.01
I ~ II	19 (19.4%)	79 (80.6%)	
III ~ IV	38 (90.5%)	4 (9.5%)	
ER			0.05
Positive	25(30.5%)	57(69.5%)	
Negative	32(55.2%)	26(44.8%)	
PR			0.09
Positive	7 (15.2%)	26 (84.8%)	
Negative	50(46.7%)	57(53.3%)	
HER-2			0.078
Positive	20(54.1%)	17(45.9%)	
Negative	37(35.6%)	66(64.4)	

### Association of EBV infection with clinicopathological characteristics

**Fig. 1:**
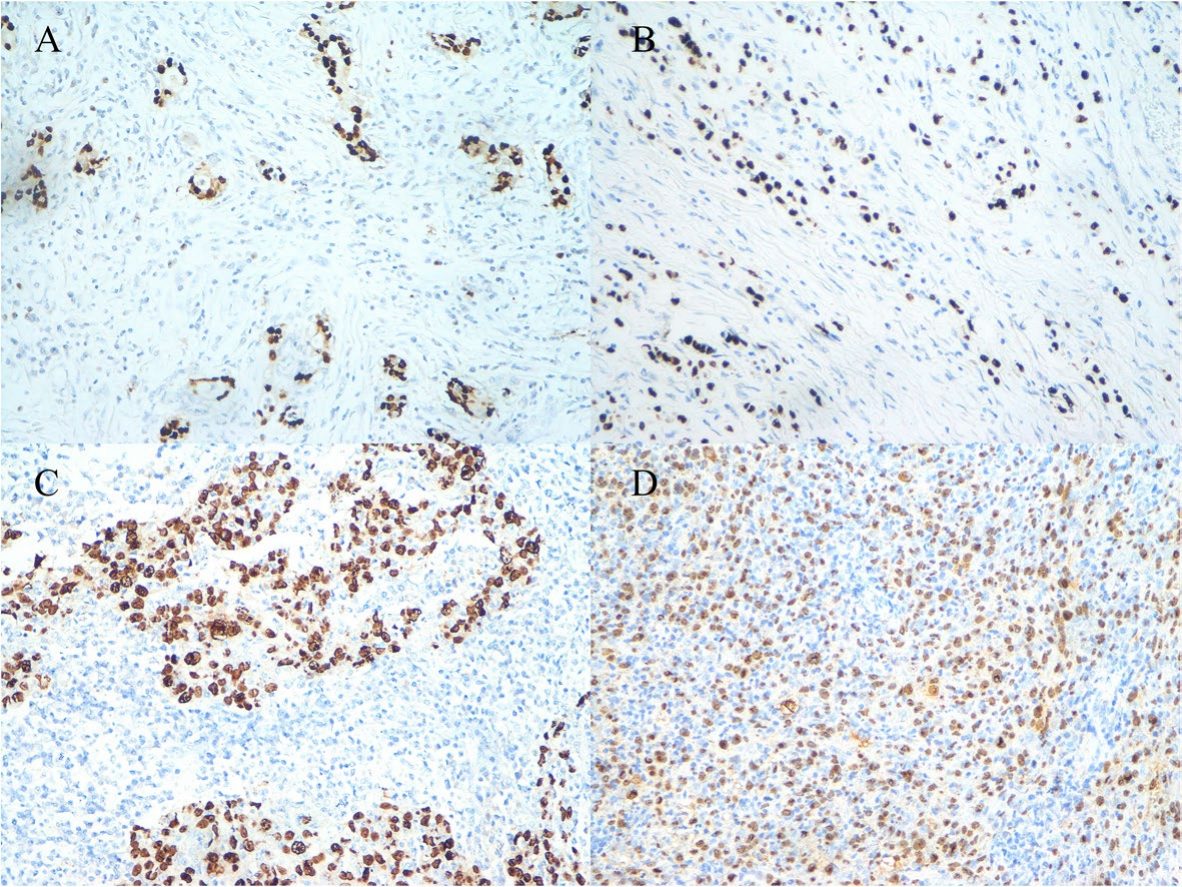
EBER-CISH technique in IBC: **A** EBER positive in invasive ductal carcinoma of grade I 200× **B** EBER positive in pleomorphic lobular carcinoma of grade I 200× **C** EBER positive in invasive ductal carcinoma of grade III 400× **D** EBER positive in invasive ductal carcinoma of grade III Magnification: 200×

Among 140 patients with IBC, EBER was detected in 57 (40.7%) IBC tissues and not detected in the adjacent tissues (*P* < 0.05). EBV infection was correlated with the tumor size, menopausal status, axillary lymph node metastasis, vascular invasion, Ki-67 index, clinical stage, and estrogen and progesterone receptor expressions (all *P* < 0.05). EBER was detected in nine (32.1%) cases of invasive lobular carcinoma, all of which were pleomorphic lobular carcinoma. Histological grades, I, II, and III were found in 7 (24.1%), 15 (42.9%), and 26 (56.3%) cases, respectively (Fig. 1; Table 1).

### Association of PD-1/PD-L1 expression with clinicopathological characteristics

**Fig. 2:**
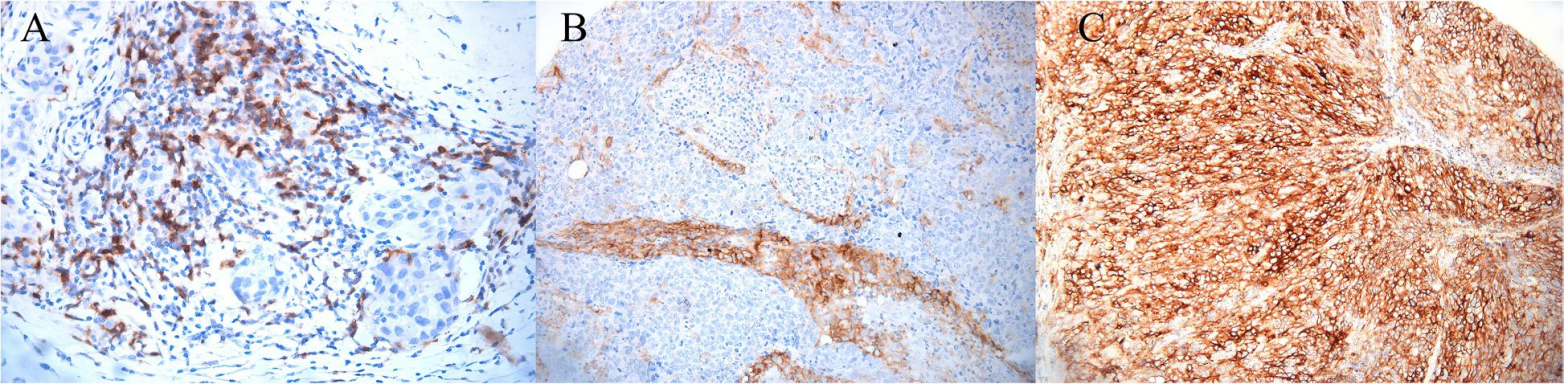
Immunohistochemistry in IBC: **A** PD-1 positive in in the stroma of IBC of grade III Magnification: 200× **B** PD-L1 positive in tumor stroma of basal cell-like breast cancer 200× **C** PD-L1 positive in basal cell-like tumor cell 400×

PD-L1 was expressed in TC, IC, and PD-1 was expressed in TILs in 23 (16.4%), 19 (13.6%), and 19 (13.6%) cases, respectively (Fig. 2). Tables 2 and 3 summarize the clinical and pathologic characteristics of all patients.

**Table 2:** Clinicopathologic characteristics of patients by PD-L1 expression

Clinicopathologic parameters	PD-L1 in TC		*P*-value	PD-L1 in IC		*P*-value
	Positive	Negative		Positive	Negative	
age			0.01			0.06
≤ 50	12(30.0%)	28(70%)		9(22.5%)	31(77.5%)	
>50	11(11.0%)	89(89%)		10(10.0%)	90(90.0%)	
tumor size			0.221			0.101
≤ 2.0 cm	11 (13.3%)	72 (86.7%)		8(9.6%)	75 (90.4%)	
> 2.0 cm	12 (21.1%)	45 (78.9%)		11 (19.3%)	46 (80.7%)	
Menopausal status			0.011			0.001
postmenopausal	10 (10.8%)	83 (89.2%)		9 (9.7%)	84(90.3%)	
menstruating	13 (27.7%)	34 (72.3%)		10(21.3%)	37(78.7%)	
Histological subtype			0.138			0.459
IDC	21(18.8%)	91(81.2%)		14(13.0%)	98(87.0%)	
ILC	2(7.1%)	26(92.9%)		5(17.9%)	23(82.1%)	
Histological classification of IDC			0.117			0.001
I	3 (10.3%)	26 (89.7%)		0(0.0%)	29(100.0%)	
II	6 (17.1%)	29 (82.9%)		4(11.4%)	31(88.6%)	
III	12 (25.0%)	36(75.0%)		10(20.8%)	38(79.2%)	
Nodal metastasis			< 0.01			0.066
Present	19(30.6%)	43(69.4%)		12(19.4%)	50(80.6%)	
Absent	4(5.1%)	74(94.9%)		7(9.0%)	71(91.0%)	
Lymphovascular Invasion			< 0.01			< 0.01
Present	18(41.9%)	25(58.1%)		14 (32.6%)	29 (67.4%)	
Absent	5(5.1%)	92(94.9%)		5 (5.1%)	92 (94.9%)	
Ki-67 Index			0.064			0.076
<14%	9(27.2%)	24(72.3%)		8(24.2%)	25(75.8)	
>14%	14(13.1%)	93 (86.9%)		11(10.3%)	96(89.7%)	
TNM Clinical Stage			< 0.01			< 0.01
I ~ II	7 (7.1%)	91 (92.9%)		6(6.1%)	92(93.9%)	
III ~ IV	16 (38.1%)	26 (61.9%)		13(31.0%)	29(69.0%)	
ER			0.001			< 0.01
Positive	6(7.3%)	76(92.7%)		2(2.4%)	80(97.6)	
Negative	17(29.3%)	41(70.7%)		17(29.3%)	41(70.7%)	
PR			0.104			0.007
Positive	2(6.1%)	31(93.9%)		0(0%)	33(100%)	
Negative	21(19.6%)	86(80.4%)		19(17.8%)	88(82.2%)	
HER-2			0.596			0.004
Positive	6(16.2%)	31(83.8%)		5(13.5%)	32(86.5%)	
Negative	17(16.5%)	86(83.5%)		14(13.6%)	89(86.4%)

**Table 3:** Clinicopathologic characteristics of patients by PD-1 expression

Clinicopathologic parameters	PD-1		
	Positive	Negative	*P-value*
age			0.01
≤ 50	7 (17.5%)	33(82.5%)	
>50	12(12.0%)	88(88.0%)	
tumor size			0.416
≤ 2.0 cm	11 (13.3%)	72 (86.7%)	
> 2.0 cm	8(14.0%)	49(86.0%)	
Menopausal status			0.171
postmenopausal	10(10.8%)	83(89.2%)	
menstruating	9(19.1%)	38(80.9%)	
Histological subtype			0.902
IDC	15(13.4%)	97(86.6%)	
ILC	4(14.3%)	24(85.7%)	
Histological classification of IDC			0.243
I	2(6.9%)	27 (93.1%)	
II	7 (20.0%)	28 (80%)	
III	6 (12.5%)	42 (87.5%)	
Nodal metastasis			< 0.01
Present	16 (25.8%)	46 (74.2%)	
Absent	3 (3.8%)	75 (96.2%)	
Lymphovascular Invasion			< 0.01
Present	16 (37.2%)	27 (62.8%)	
Absent	3 (3.1%)	94 (96.9%)	
Ki-67 Index			0.019
<14%	9 (27.2%)	24(72.8%)	
>14%	10(9.3%)	97(90.7%)	
TNM Clinical Stage			< 0.01
I ~ II	6 (6.1%)	92 (93.9%)	
III ~ IV	13 (31.0%)	29 (69.0%)	
ER			0.047
Positive	7(8.5%)	75(91.5%)	
Negative	12(25.0%)	46(75.0%)	
PR			0.161
Positive	2(6.1%)	31(93.9%)	
Negative	17(15.9%)	90(84.1%)	
HER-2			0.596
Positive	6(16.2%)	31(83.8%)	
Negative	17(16.5%)	86(83.5%)	

### Correlation between the status of EBV infection and PD1/PDL1

EBV infection was correlated with PD-L1 and PD-1 expressions. The positive rate of PD-1/PD-L1 expression was significantly higher in the EBV-positive group than in the EBV-negative group (Table 4).

**Table 4:** Correlation between the status of EBV infection and PD1/PDL1

	IBC tissue EBER-ISH (+)	IBC tissue EBER-ISH (−)	*P*-value
PD-L1(+) in TC	18 (78.3%)	5 (21.7%)	< 0.01
PD-L1(−) in TC	39 (33.3%)	78 (66.7%)	
PD-L1(+) in IC	16 (84.2%)	3 (15.8%)	< 0.01
PD-L1(−) in IC	41 (33.9%)	80 (66.1%)	
PD-1 (+)	17 (89.5%)	2 (10.5%)	< 0.01
PD-1 (−)	40 (33.1%)	81(66.9%)	

### Survival analysis

**Fig. 3:**
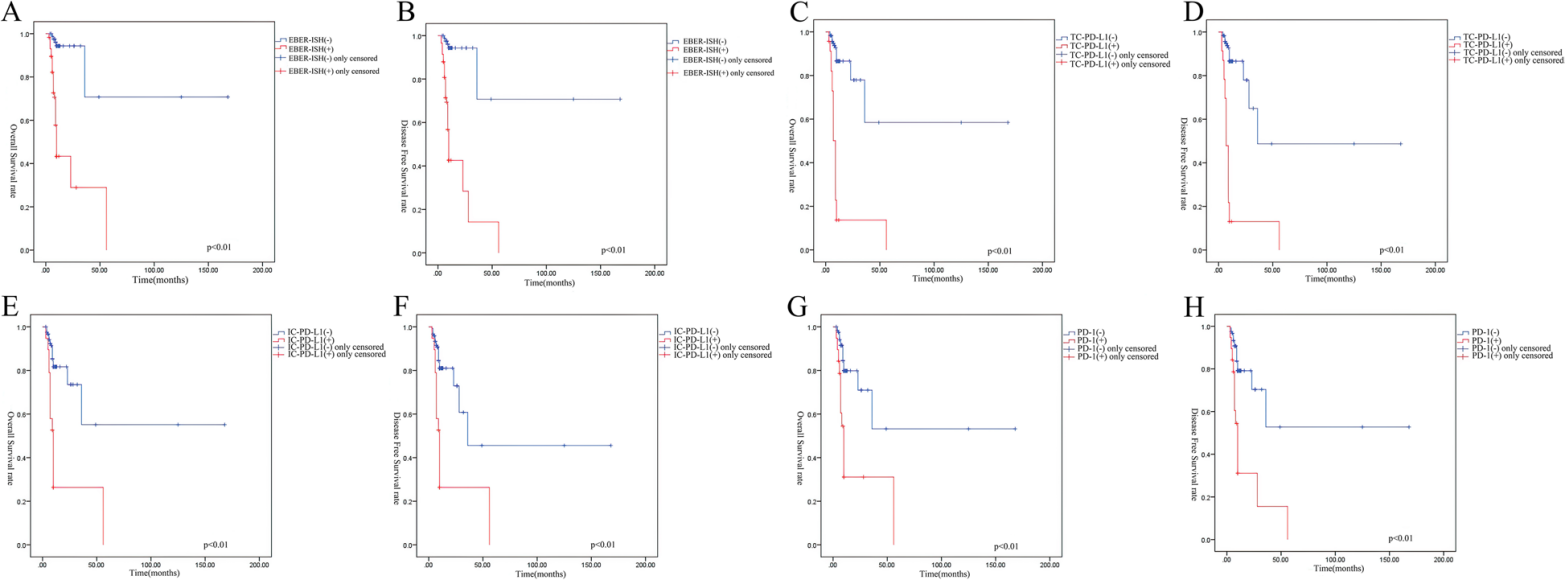
**A&B** Kaplan–Meier estimates of DSF and OS of IBC patients according to IBC tissue EBER-ISH; **C&D** Kaplan–Meier estimates of DSF and OS of IBC patients according to TC-PD-L1; **E&F** Kaplan–Meier estimates of DSF and OS of IBC patients according to IC-PD-L1; **G&H** Kaplan–Meier estimates of DSF and OS of IBC patients according to PD-L1

**Fig. 4:**
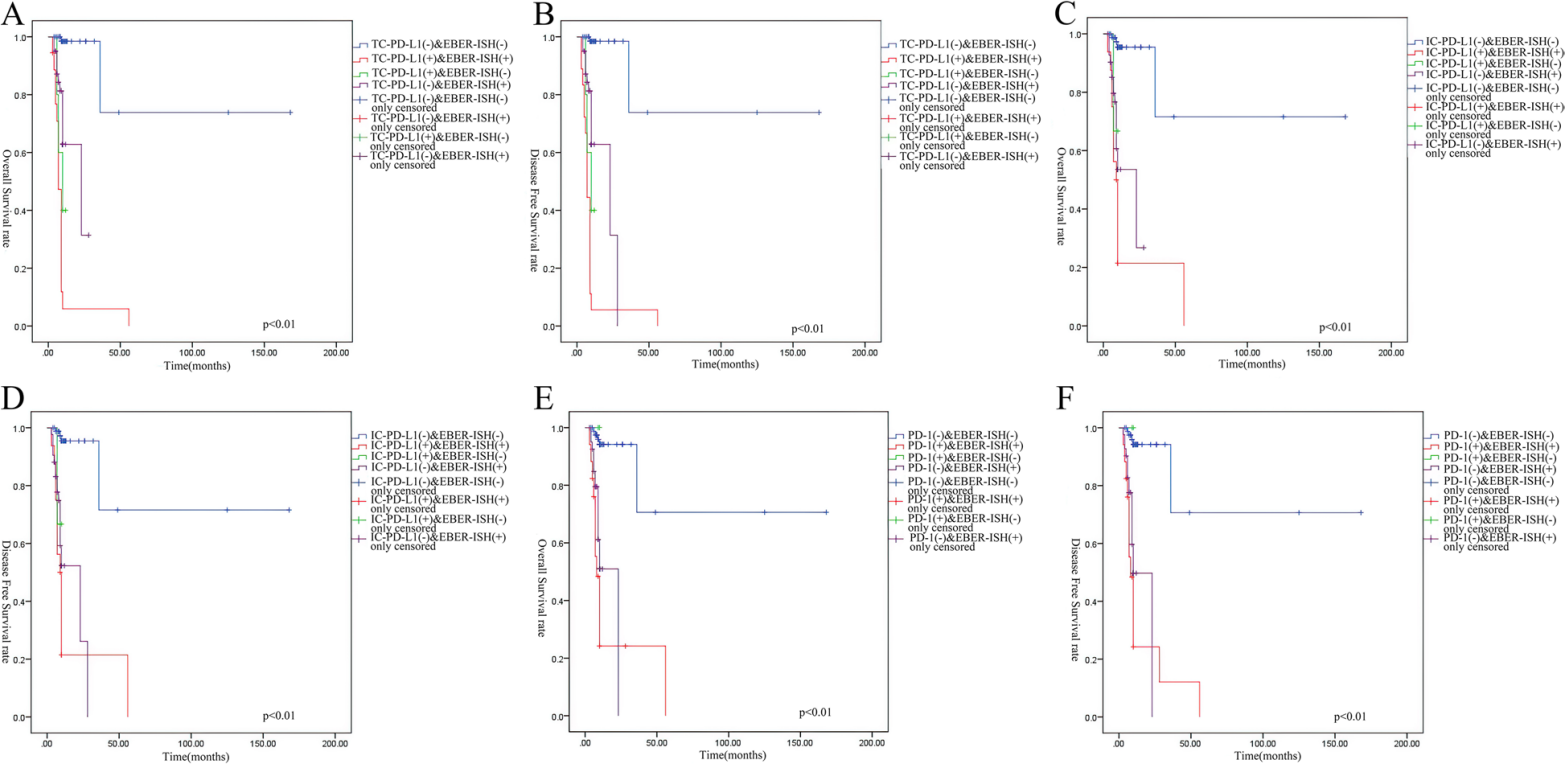
**A&B** Kaplan–Meier estimates of DSF and OS of double positive for EBV and TC-PD-L1 IBC patients compared to cases that were double negative or single positive IBC patients. **C&D** Kaplan–Meier estimates of DSF and OS of double positive for EBV and IC-PD-L1 IBC patients compared to cases that were double negative or single positive IBC patients. **E&F** Kaplan–Meier estimates of DSF and OS of double positive for EBV and PD-1 IBC patients compared to cases that were double negative or single positive IBC patients

The last follow-up was in May 2020, with a total of 35 patients experiencing an endpoint event. The Kaplan–Meier univariate survival analysis showed that EBV infection was associated with poor disease-free survival (DFS) and overall survival (OS) in patients with IBC (logrank *P* < 0.01; Fig. 3A-B). Kaplan–Meier estimates of DFS and OS of patients with IBC according to PD-L1/PD-1 expression showed that positive PD-L1/PD-1 expression could also predict a worse prognosis (logrank *P* < 0.01; Fig. 3C-H). The survival analysis of cases positive for both EBV infection and PDL1/PD1 expression showed the worst DFS and OS among all groups (logrank *P* < 0.01; Fig. 4A-F).

## Discusion

In 2020, breast cancer ranked first in the incidence of cancers worldwide, with approximately 2.3 million new cases every year [19]. Breast cancer is a multifactorial disease. Infectious factors account for approximately 18% of all cases of cancer worldwide [20]. EBV, the first identified human tumor virus, is widely spread in the human population and causes various infectious diseases [21]. It is the member of the human γ-herpesvirus family. EBV infection is characterized by lifetime latent infection [22]. BV exists widely in the human body and promotes the transformation of human cells, leading to unlimited cell proliferation. It is associated with various lymphomas, such as Burkitt’s lymphoma and Hodgkin lymphoma, and various solid tumors, such as nasopharyngeal and gastric cancers. EBV can also cause other diseases, such as lymphoproliferative disease after transplantation and autoimmune disease. Since Labrecque et al. first reported the correlation between EBV and breast cancer in 1995 [6], the correlation between EBV and breast cancer has been studied extensively, but the results are controversial [23–26].

Labrecque et al. detected EBV infection in 19 of 91 (21%) cases of breast cancer by polymerase chain reaction (PCR) [6]. Murray et al. assessed EBV nuclear antigen 1 (EBNA1) in 153 cases of breast cancer by immunohistochemistry and found that the monoclonal antibody 2B4–1 was positive in 47 (31%) cases [27]. Highly sensitive quantitative real-time PCR showed a low EBV DNA level. A recent meta-analysis indicated a significant association of EBV infection with the breast cancer risk (odds ratio [OR] = 4.75; 95% confidence interval [CI]: 2.53–8.92; *P* < 0.01) with significant heterogeneity (I2 = 65.3%) [28]. However, Kadivar et al. found that none of the 100 breast cancer samples or 42 control specimens from Iranian women showed EBV DNA by PCR [23]. Moreover, Dowran et al. found that none of the cancerous or benign samples from Iran were positive for EBV by PCR [26]. The different results may be due to the limitation of sample sources or geographic differences in the frequency of EBV infection and biopsies [25, 28]. Therefore, the detection rate differed with sample composition, study methodology, and ethnicity of patients. Another study detected 84 cases of EBV infection in 300 Chinese patients with breast cancer, with a positive detection rate of 28.00% in breast cancer and 71.43% in overall cancer [29]. We also reviewed the big data of Chinese patients with EBV infection. In many cities in China, EBV infection was found in over 80% of children above 6 years old and 90% of children above 8 years old. EBV can easily spread through saliva. In China baby feeding habits included mouth feeding or prechewing food to feed babies, which lead to early EBV infections [30]. According to another survey, approximately 98% of Chinese people were infected with EBV before 30 years of age [31]. Because cellular and humoral immune functions are decreased in the elderly patients, and the proportion of patients with EBV-related tumors is high [32]. The elderly patients were high in proportion in this cohort. Patients with a history of cancer are also immunocompromised, making them susceptible to EBV infection. In our study, 13 patients had cancer, including thyroid, gastric, and lung cancers, before breast cancer was detected, and eight of them were positive for EBV infection. CISH showed that EBV infection was present in some IBC infection. EBER was detected in 57 of 140 (40.7%) patients with IBC.

In this study, the EBER detection rate was correlated with the tumor diameter, menopausal status, axillary lymph node metastasis, lymphovascular invasion, Ki-67 index, molecular type, and ER and PR expressions and not correlated with the histological grade or type of breast cancer. However, it increased with an increasing histologic grade. The incidence of high-grade IBC was higher among patients with EBV infection than in those without. EBER expression was also associated with a poor prognosis. Patients with EBV infection were more prone to lymph node metastasis (59.7%) and lymphovascular invasion (72.1%) and presented with a worse clinical stage of patients, mostly III or IV (90.5%). These results suggest that IBC presents with more aggressive characteristics in patients with EBV infection, consistent with Mazouni et al.’s study [33]. Naby et al. found that nodal involvement was associated with EBV infection [34]. Nagi et al. found that EBV infection correlated with the tumor grade in breast cancer [35]. EBV-positive breast cancer was associated with elevated ER and PR expressions but not HER2 expression. Mazouni et al. found EBV-positive breast cancer had a more aggressive phenotype and was more frequently negative for ER expression [33]. Lin et al. demonstrated that EBV-encoded BARF0 promoted the tumorigenic activity of breast cancer cells by activating HER2/HER3 signaling cascades [36]. As a nuclear antigen expressed by proliferating cells, Ki-67 is a reliable indicator for the proliferative activity of tumor cells. The critical value distinguishing high and low Ki-67 expressions range from 3.5 to 34%. In 2011, at the St. Gallen International Breast Cancer Conference, the critical value for positive Ki-67 expression was determined as 14%, and the treatment plan of early breast cancer would be formulated accordingly [35]. Although the results may vary because of the different detection methods of Ki-67 expression at different laboratories, clinically, 14% is the commonly used critical value for Ki-67 expression, as in our study. Ki-67 protein expression is closely related to the pathological characteristics of breast cancer and can be used as an indicator of high-grade malignancy. In our study, 50 of the 57 EVB-positive patients showed Ki-67 expression, suggesting that breast cancer presents with more aggressive features in patients with EBV infection.

The potential mechanism of EBV promoting breast cancer has been studied. EBV can infect primary mammary epithelial cells expressing CD21, which alters gene expression and stimulates the oncogenic signaling via c-Met. When mammary epithelial cells were implanted as xenografts, EBV infection cooperated with activated Ras and accelerated the formation of breast cancer [37]. Methylation detection for EBV-related breast cancer has shown that epigenetic detection could demonstrate the occurrence of breast cancer [38]. Abdallah et al. identified TP73 and TBX15 as breast cancer biomarkers and significantly enriched developmental pathways, including Hippo and Wnt signaling pathways. The bioinformatics analysis indicated a possible role of EBV infection in breast cancer tumorigenesis. Human papillomavirus and EBV can coexist in breast cancer and may play a role in its development. Oncoproteins (E6/E7, latent membrane protein 1 [LMP1], and EBNA1) of high-risk human papillomavirus and EBV can interact with each other via the epithelial–mesenchymal transition to cause initiation and/or progression of human breast cancer [39]. Thus, EBV infection promotes the occurrence and progression of breast cancer. In this study, patients were followed-up from the day of surgery to May 2020, with distant metastasis and/or death as endpoints. A total of 32 patients experienced an endpoint event, 29 of whom were EBV-positive. The Kaplan–Meier univariate survival analysis showed that the DFS was longer in EBV-negative patients than in EBV-positive patients (χ2 = 47.376, *P* < 0.01). Tsai et al. showed that EBV affected OS. Viral infection-associated breast cancer has a poor prognosis, particularly when multiple viruses affect breast tissue [40].

Since the prognosis of EBV-positive breast cancer is poor, novels treatments should be explored for these patients. The conventional treatment methods of breast cancer include surgery, chemotherapy, radiotherapy, and endocrine therapy. Further, immunotherapy can treat advanced solid tumors by regulating the host’s antitumor immune response and enhancing the autoimmune function to recognize and eliminate tumor cells. In recent years, immune checkpoint inhibitors, including PD-1/PD-L1 inhibitors, have been investigated. The function of the PD-L1/PD-1 signaling pathway is reversible. When PD-L1 interacts with the PD-1 receptor, the immune response of T lymphocytes is inhibited, and the tumor cells escape the immune system [41]. Blocking the PD-L1/PD-1 interaction can enhance the immune function of T lymphocytes and exert an antitumor effect. These results have been verified in animal tumor models. In some national clinical trials (NCT01042379, NCT02819518), compared to the control group, the PD-1/PD-L1 inhibitor with chemotherapy increased the pathologic complete response rate and prolonged progression-free survival [42, 43]. In various cancers related to EBV infection, PD-L1/PD-1 expression is increased [4, 10–12]. The upregulation of PD-L1 expression is related to EBV infection [10–12]. Whether or not this phenomenon exist in breast cancers infected with EBV is unknown.

We found that there were 23 cases (16.4%) with expression of PD-L1 in TC in and 19 cases (13.6%) in IC. There were 19 cases (13.6%) with PD-1 expression in tumor stroma. EBV infection was correlated with PD-L1 and PD-1 expressions. The positive rate of PD-1/PD-L1 expression was significantly higher in the EBV-positive group than that in the EBV-negative group. PD-L1 expression could be upregulated in EBV-infected breast cancer, leading to the promotion of tumor progression and poor prognosis. Similarly, PD-L1 expression is elevated in EBV-infected tumors. Fang et al. proposed that PD-L1 expression increased in EBV-infected nasopharyngeal carcinoma [44]. EBV-induced exogenous or excessive endogenous LMP-1 can significantly increase and upregulate PD-L1 expression, such as in nasopharyngeal carcinoma associated with EBV infection. Elevated LMP-1 significantly increases the activities of signal transducer and activator of transcription (STAT) 3, nuclear factor kappa B, and nuclear transcription factor activator protein 1; participates in cell proliferation and anti-apoptosis; and regulates the expression levels of key proteins that induce invasion, leading to the occurrence of tumors [44]. In 2014, the cancer genome mapping program divided gastric cancer into four gene subtypes, including EBV-positive gastric cancer, or “EBVaGC.” [45]. In a previous study, PD-L1 overexpression occurred in more than half of the cases of EBV-infected gastric cancer [46]. Inflammatory factors are upregulated by antitumor and/or antiviral immune response(s). Tumor cells can use these immune responses to escape immune surveillance. Interferon-γ is the most common inflammatory factor regulating PD-L1 expression [47]. It can lead to PD-L1 overexpression by activating the Janus kinase 2/STAT1/interferon regulatory factor 1 signaling pathway in EBVaGC [46]. A study on EBV-related classic Hodgkin lymphoma showed many CD4+ and CD25+ regulatory T cells in tumor microenvironment. The increased PD-L1 expression may promote the generation of regulatory T cells. However, this phenomenon was not found in classic Hodgkin lymphoma without EBV infection. In this study, PD-L1/PD-1 expression could also predict a poor prognosis. These patients should undergo combined multimodality treatment for enhanced antitumor effect. Although this study found that breast cancer with EBV infection was related to high PD-L1 expression, the mechanism should be explored in future studies.

We used the data of 140 patients to analyze the correlation between PD-1/PD-L1 expression and clinicopathological features of IBC and found that PD-L1 expression in TC was related to the age, menopausal status, lymph node metastasis, lymphovascular invasion, TNM clinical stage, and ER expression (all *P* < 0.05). PD-L1 expression in IC was related to the menopausal status, lymph node metastasis, lymphovascular invasion, TNM clinical stage, and ER, PR, and HER2 expressions (all *P* < 0.05). PD-1 expression was associated with the age, lymph node metastasis, lymphovascular invasion, Ki-67 index, TNM clinical stage, and ER and PR expressions (all *P* < 0.05). Zhang et al. performed a systematic search of PubMed, EMBASE, and Cochrane Library databases to determine the correlation of PD-L1 expression with clinicopathological features [48]. PD-L1 expression was associated with positive lymph node metastasis, higher histological grades, ER-negativity, and triple-negative breast cancer. A review concluded that PD-L1 expression was heterogeneous and generally associated with the presence of tumor-infiltrating lymphocytes and poor prognostic factors, such as young age, high tumor grade, ER-negativity, PR-negativity, and HER-2 overexpression [49].

In this study, EBV-infected IBC showed a large tumor mass, postmenopausal status, easy occurrence of axillary lymph node metastasis and lymphovascular invasion, and higher clinical stage. Patients with EBV-positive breast cancer were more likely than those with EBV-negative breast cancer to develop high PD1 and PDL1 expressions. As proposed by some scholars, the term “Epstein–Barr virus-associated pulmonary carcinoma” is used for lymphoepithelioma-like lung cancer [50].

Whether or not patients with EBV-related tumors would benefit from tumor immunotherapy targeting PD-L1/PD-1 should be studied in the future with specific regulation mechanism of PD-L1 expression in EBV-infected tumors. Novel treatments and indications of immunotherapy should be further explored.

## Data Availability

The datasets used and/or analyzed during the current study are available from the corresponding author on reasonable request.
